# Nutritional Characterization of Fruits from Three African Plant Species: *Dialium guineense* Willd, *Parkia biglobosa* Jacq. and *Andansonia digitata* L.

**DOI:** 10.3390/plants14152344

**Published:** 2025-07-29

**Authors:** Manuela Lageiro, Jaime Fernandes, Ana C. Marques, Manuela Simões, Ana Rita F. Coelho

**Affiliations:** 1 INIAV, Instituto Nacional de Investigação Agrária e Veterinária IP, 2784-505 Oeiras, Portugal; jaime.fernandes@iniav.pt; 2 Earth Sciences Department of NOVA School of Sciences and Technology, Campus de Caparica, 2829-516 Caparica, Portugal; amc.marques@campus.fct.unl.pt (A.C.M.); mmsr@fct.unl.pt (M.S.); 3 GeoBioTec Research Center, NOVA University Lisbon, 2829-516 Caparica, Portugal

**Keywords:** African indigenous fruits, pulp and seed, physicochemical composition, elemental analysis, amygdalin, colour, energy

## Abstract

*Dialium guineense* (velvet tamarind), *Parkia biglobosa* Jacq. (African locust bean) and *Adanosonia digitata* L. (baobab) are fruits from African plants whose nutritional potential remains poorly characterised. As such, their pulps and seeds were analysed for colour (CIELab system), moisture, ash, protein, fat, soluble and insoluble dietary fibre, free sugars (HPLC-RI), organic acids (HPLC-PDA), macro and microelements (XRF analyser) and amygdalin (HPLC-PDA). The colours of their pulps differed considerable (ΔE > 38 between the velvet tamarind and African locust bean) and the moisture content was lower in seeds (about 7%) compared to pulps (9–13%). Seeds were more concentrated in protein (20–28%) and fat (5–22%), whereas pulps were richer in sugar (1–12%). African locust bean pulp was the sweetest (39% total sugar), while baobab pulp contained the highest soluble fibre (>30%) and citric acid (3.2%), and velvet tamarind pulp was distinguished by its tartaric acid content (3.4%). Seeds of the African fruits presented higher Ca, P, S and Fe contents, whereas pulps had higher K content. No amygdalin (<6.34 mg per 100 g of dry weight) or toxic heavy metal contents were detected. The PCA segregated samples by pulp and seed and the PC1 explains the sugar and moisture of the pulps, while protein, fat and minerals are associated with the seeds. These data confirm that African fruit pulps and seeds have distinct functional profiles, are safe for food use and can be consumed, which is important for efforts to promote the conservation of these tropical plant species.

## 1. Introduction

Growing concerns about global food security and chronic malnutrition, especially in tropical regions of developing countries such as sub-Saharan Africa, have driven the demand for sustainable and nutritionally rich food sources that can meet the emerging needs of populations. In this context, and following FAO guidelines, native African wild fruits play a strategic role as alternative food resources, both due to their local availability and their still undervalued nutritional potential. Despite their traditional use in food and folk medicine, many of these species remain neglected from a scientific perspective, which limits their agri-food, economic, and functional applications [1,2,3,4].

Among the endogenous fruits of West and sub-Saharan Africa with nutritional and cultural relevance, the following stand out: *Dialium guineense* Willd. (velvet tamarind), *Parkia biglobosa* Jacq. (African locust bean) and *Adansonia digitata* L. (baobab). These fruits are consumed both fresh and processed into juices, sauces, flours or medicinal preparations. The pulp is generally the most valued part, while the seeds, often discarded, represent a by-product with high potential for nutritional and economic value. The fruits and seeds of the studied species are potential sources of essential nutrients including proteins, carbohydrates, fibres, minerals, and bioactive compounds [3].

Velvet tamarind, a member of the *Fabaceae* family, specifically the subfamily *Detarioideae*, is a medium-sized tree that produces a small, dark brown drupe with a thin, brittle skin and a sticky, acidic, sweet pulp that varies in colour from orange to reddish-brown, containing 1 to 2 hard seeds. Its pulp, fresh or dried, is used to make soy sauce, a condiment, or eaten as a delicacy. It is also used in traditional medicine as an antidiarrheal and antipyretic [5,6,7].

African locust bean, from the *Fabaceae* family, subfamily *Mimosoideae*, is a large tree with a broad, rounded crown and spherical inflorescences in hanging flower heads pollinated by bats. Its fruit is a long, flat pod, 10–30 cm long, containing multiple seeds immersed in a sweet, floury yellow pulp. Its seeds are hard and dark brown in colour. Its pulp is used to make sweets and flours, while its seeds can be fermented and used to make a traditional condiment with a high protein content (*dawadawa*). It also has medicinal uses among indigenous peoples [8,9,10,11].

Baobab, from the *Malvaceae* family, subfamily *Bombacoideae*, is a monumental tree that can reach heights of 20–30 m and trunk diameters of 10–14 m. It has cultural relevance for African peoples and is nicknamed the “tree of life”. It has large, white, hanging flowers that are pollinated by bats. Its fruit has a woody capsule, ovoid to oblong in shape, covered by a velvety shell. Its interior is dry, with a white to beige pulp that has a sour taste and covers the seeds. Its pulp is used to produce juices, flours and supplements; its ground seeds are used to extract oil and produce functional flours [12,13]. Of the three fruits, baobab is the most extensively studied and has spread beyond its native habitat. Its greater recognition as a food with potential led to its addition to the list of novel food components in the European Union in 2008. The following year, the Food and Drug Administration also recognised it as a food ingredient [14,15].

**Figure 1:**
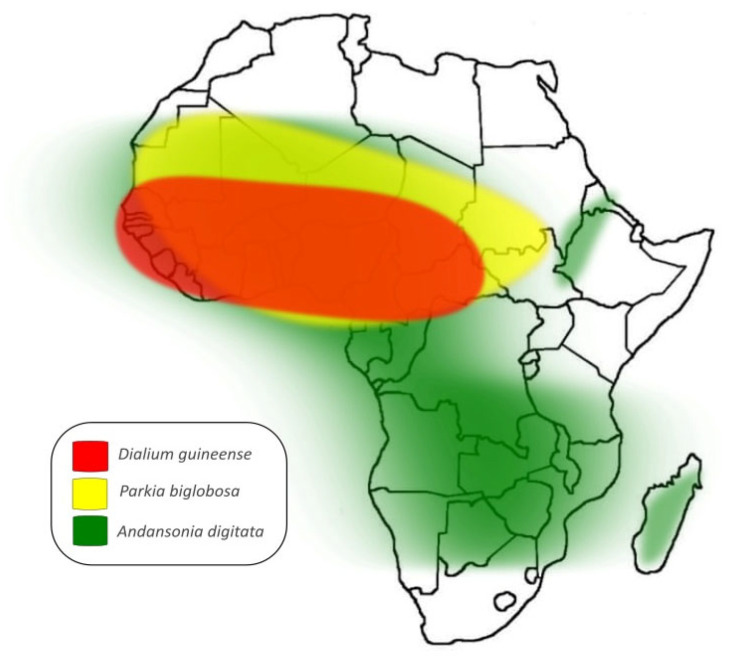
Distribution of the plant species under study in their native African continent (made by the authors).

Figure 1 illustrates the distribution of the three plant species in their native context on the African continent.

Although some articles can be found, the scientific information on the composition of these fruits and their food safety, and particularly the distinction between their pulp and seeds, is scarce, fragmented and few make a systematic assessment, particularly with regard to the fibre content, soluble and insoluble; organic acid profile; free sugar profile; elemental composition; energy value and potential toxicity.

The lack of such data limits the integration of these fruits into strategies for food diversification, product reformulation and the development of new functional ingredients. Furthermore, the comparative characterisation of pulps and seeds is essential for exploring new technological applications, particularly in the use of seeds as raw materials for flours or supplements rich in minerals and fibres. Baobab is the only fruit for which studies have been conducted on the integration of its flours, for example, in cakes [16] and chocolates [17].

Thus, the present study aimed to perform a comprehensive physicochemical and nutritional characterisation of the pulps and seeds of velvet tamarind, African locust bean and baobab. Parameters such as colour, moisture, ash, protein, fat, soluble and insoluble dietary fibre, free sugars, organic acids, mineral content (Ca, K, P, S, Fe, Cu), and energy value were evaluated.

Additionally, the presence of amygdalin in the seeds was quantified to assess the toxicological risk associated with their consumption. The data presented in this article focus on the physicochemical and nutritional characterisation of the pulps and seeds of *Dialium guineense* (velvet tamarind), *Parkia biglobosa* Jacq. (African locust bean), and *Adansonia digitata* L. (baobab), evaluating the compositional differences between these two fractions and their functional potential. Additionally, the safety of consuming the seeds is considered, particularly regarding the presence of potentially toxic compounds, such as amygdalin. This study could serve as a starting point for creating strategies to valorise by-products, develop functional ingredients, and formulate foods tailored to specific nutritional contexts, by providing robust and comparative data on the nutritional composition of these three tropical fruits with recognised traditional uses. This study contributes to filling a relevant gap in the literature and reinforces the scientific appreciation of native plant species with high functional and economic potential.

## 2. Results

### 2.1. Colourimetric Analysis

**Figure 2:**
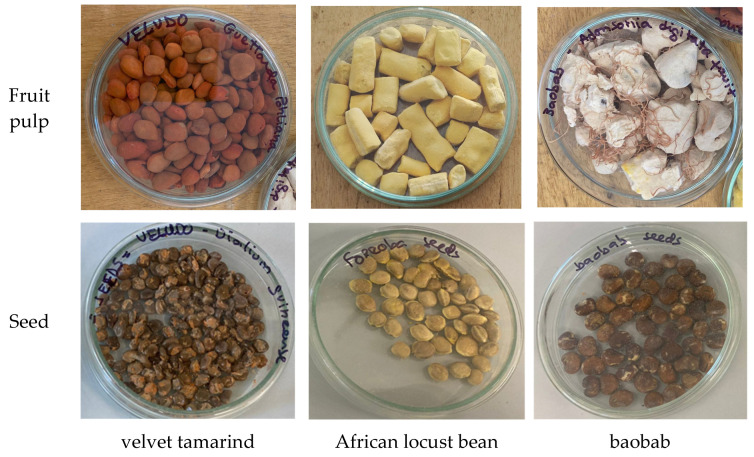
Pictures of the fruit pulps and seeds of *Dialium guineense* Willd (velvet tamarind), *Parkia biglobosa* Jacq. (African locust bean) and *Andansonia digitata* L. (baobab).

In Figure 2 are presented the visual appearance of the pulps and seeds of the three African fruits: *Dialium guineense* Willd (velvet tamarind), *Parkia biglobosa* Jacq. (African locust bean) and *Andansonia digitata* L. (baobab).

Colourimetric analysis was carried out for pulps and seeds of the different tropical fruits (velvet tamarind, African locust bean and baobab) (Table 1). Regarding the L* parameter, the pulp of the African locust bean is the lightest (L* = 79.9), while velvet tamarind showed the darkest pulp. In contrast, the velvet tamarind seed is lighter than the pulp. For the a* parameter, a significantly higher value was obtained for velvet tamarind pulp, while the lowest was obtained for both pulp and seed of African locust bean, having a more greenish colour. As such, the pulp of African locust bean is more yellow, showing a significantly higher value (b* = 38.4). In all three fruits, the seeds presented a lower b* compared to the pulp. Considering the whiteness index (WI) it is possible to verify that the pulp of African locust bean is whiter and the pulp of velvet tamarind darker according to the L* parameter. Regarding the saturation (C_ab_*), the pulp of African locust bean has a huge saturation regarding the yellows and the seed of velvet tamarind showed lower saturation. African locust bean showed similar hue angle values for seed and pulp, despite being significantly different, having a yellowish hue angle, while velvet tamarind has a reddish-brown hue angle and baobab has a yellow–orange hue angle.

Considering Table 2, the total colour difference analysis revealed variations among both the fruits’ species and their parts (pulp and seed). In fact, the most notable difference can be observed between velvet tamarind and African locust bean pulps (ΔE = 37.99), indicating highly distinct visual appearances between these two pulps. Considering the seeds, the highest difference was observed between African locust bean and baobab, suggesting substantial pigment variations regarding seeds, followed by velvet tamarind and baobab. Regarding the contrast between pulp and seed, the greatest colour difference was observed between velvet tamarind and African locust bean, with a ΔE of 28.84, reinforcing the difference in colour for both parts (pulp and seed), while the lowest contrast regarding colour difference was obtained between African locust bean and baobab with a ΔE of 5.10. As such, the data obtained shows the difference between each fruit and each part (pulp and seed).

### 2.2. Moisture, Ash, Protein, Fat and Dietary Fibre Content

The analysis of moisture, ash, fat and fibre content of the different fruits (Table 3) revealed variations regarding their contents in pulps and seeds. Moisture content showed higher values (i.e., water content) in African locust bean pulp and the lowest in this fruit’s seed. In fact, in all three tropical fruits analysed, moisture content was lower in seeds compared to pulp. Regarding ash content, the values ranged between 2.04 to 2.24% in fruit pulps and between 2.38 and 2.53% in fruit seeds, showing a higher content in seeds relative to pulps. Protein content was significantly lower in pulps (especially in baobab), showing higher values in seeds. Indeed, African locust bean pulp showed the highest protein content, with an average of 28.41%. Regarding fat content, as observed for protein content, the seeds showed a significantly higher content of fat relative to pulps, presenting the highest content in Baobab seeds, 21.5% fat.

**Figure 3:**
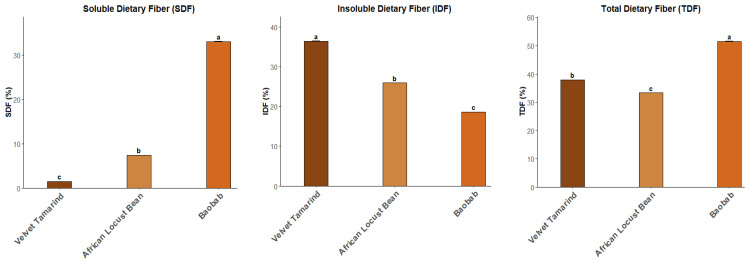
Dietary fibre (insoluble (IDF), soluble (SDF) and total (TDF) (% of dry weight)) of fruit pulps of *Dialium guineense* Willd (velvet tamarind), *Parkia biglobosa* Jacq. (African locust bean) and *Andansonia digitata* L. (baobab). The data are presented as the means ± SDs (*n* = 3). ANOVA, *p* < 0.05, performed for each parameter. Different letters express significant differences between each type of fruit (a, b), with a for the highest values.

The pulp of the three tropical fruits were analysed regarding dietary fibre percentages (soluble dietary fibre (SDF), insoluble dietary fibre (IDF) and total dietary fibre (TDF) (Figure 3) and significant differences were observed among African locust bean, baobab and velvet tamarind. Baobab showed significantly higher values of SDF and lower IDF, being the opposite verified for velvet tamarind. The African locust bean showed the lowest content of total dietary fibre compared to the other fruits.

### 2.3. Organic Acid Profile

Organic acid profiles of both pulps and seeds of the three tropical fruits are presented in Table 4. The baobab pulp showed the highest content of acetic acid, followed by a significantly lower concentration in velvet tamarind seeds. The citric acid in the pulps of both African locust bean and baobab were similar (equal in terms of statistics) and the seeds of the three tropical fruits showed lower values—about 70% lower. For both formic and oxalic acids, the highest content was observed in baobab pulp, while for malic and succinic acids the highest contents were obtained in velvet tamarind seeds and in African locust bean pulp, respectively. For lactic acid, only the pulps of baobab and velvet tamarind were quantified, showing lower and not significantly different values. Regarding tartaric acid, the only value quantified was from velvet tamarind pulp, with a value of 3.43% of the sample (on a dry weight basis). Overall, for the majority of the organic acids analysed, the pulp showed a higher content than the seeds and the baobab pulp had higher contents of acetic, citric, and lactic acids, indicating an acid characteristic of this fruit’s pulp.

### 2.4. Free Sugars Profile

Sucrose, glucose, fructose and total sugar contents were quantified in the pulps and seeds of velvet tamarind, African locust bean and baobab, which were previously assessed in Lageiro et al. [18]. Among the pulps, African locust bean is by far the sweetest (38.80% of total sugar), whereas baobab pulp contains the lowest sugar content (6.53% of total sugar). In contrast, among seeds, there were no significant differences regarding the three fruits, showing values below 3% for total sugar content. Overall, the highest concentration of sugar was in the pulps, and sucrose was the predominant carbohydrate, followed by glucose and fructose (sucrose > glucose > fructose). In the pulps of the three fruits, the values of sucrose, glucose, fructose and total sugar varied between 1.99–15.50%, 2.12–12.07%, 1.72–9.89%, and 6.53–38.80%, respectively. In seeds, the values ranged between 0.40–1.45%, 0.23–0.45%, 0.23–1.04%, and 0.86–2.96%, respectively, for sucrose, glucose, fructose and total sugar content. The data relative to the free sugars’ profile is essential to understand relationships among the different parameters studied, being analysed and studied in Section 2.8.

### 2.5. Carbohydrates and Energy

Table 5 presents the carbohydrate contents (calculated by Equation (5)) and energy (calculated by Equation (6)) of velvet tamarind, African locust bean and baobab fruit pulps. The results indicate that African locust bean had a significantly higher value of carbohydrates and energy, while baobab showed the lowest values. As such, there is a tendency of carbohydrate contents and energy of African locust bean > velvet tamarind > baobab.

### 2.6. Element Contents

Calcium (Ca), potassium (K), phosphorus (P), sulfur (S), iron (Fe) and copper (Cu) contents were quantified in both pulps and seeds of velvet tamarind, African locust bean and baobab fruits (Table 6), showing significant differences among samples. The results demonstrated that Ca and Fe presented a higher content in seeds, whereas the K and Cu contents are predominant in the pulps. For instance, velvet tamarind seeds showed the highest Ca content and baobab seeds can be considered the highest Fe source, with triple the content found in any of the pulps. Also, baobab pulp showed the highest amount of K, while the pulp of African locust bean showed the highest Cu content. Also, baobab seeds exhibited the highest P and S among all samples, with values much higher than the ones obtained for pulps. Overall, regarding the three tropical fruits, the seeds are superior sources of Ca, Fe, P and S, while the pulps are better sources of K.

### 2.7. Toxicological Analysis

No toxic levels were quantified for the elements analysed (Hg, Pb, As, Ni, and Cd was <20 mg·kg^−1^).

Amygdalin, a toxic compound present mostly in Prunus fruit seeds, can be metabolised to cyanide in the human body. Cyanide is a highly toxic compound that interferes with the cells’ ability to utilise oxygen in sufficiently high doses, leading to symptoms of cyanide poisoning such as dizziness, weakness, difficulty of breathing and, in extreme cases, convulsions and death. The reaction involves the β-glucosidase enzyme, which breaks down the amygdalin into glucose, cyanide and benzaldehyde.

Amygdalin was not detected in the studied fruit seeds. The method limit of detection was 6.34 mg of amygdaline per 100 g of dry sample, so the seeds had less than this limit of detection concentration.

### 2.8. Principal Component Analysis

**Figure 4:**
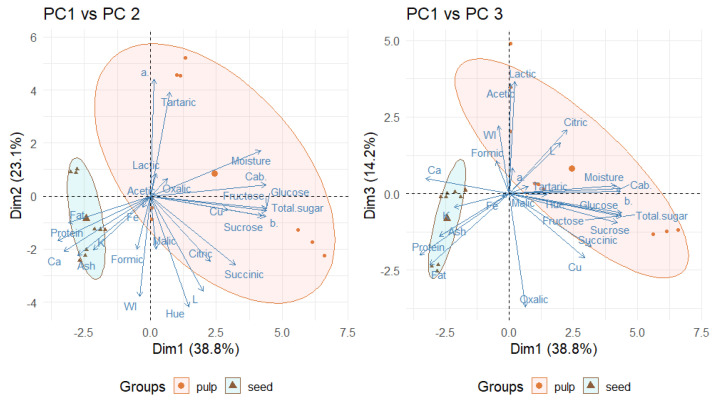
Principal component analysis (PCA) for PC1 vs. PC2 and PC1 vs. PC3 for each parameter studied (variance of 38.8% for PC1, 23.1% for PC2 and 14.2% for PC3) with an eigenvalue of 10.1 for PC1, 6.0 for PC2, and 3.7 for PC3, with a total variance of PC1, PC2, and PC3 of 76.01%.

The principal component analysis (PCA) was performed (Figure 4) to evaluate the overall distribution of the fruit samples based on the parameters analysed. As such, Figure 4 shows two biplots: PC1 vs. PC2 and PC1 vs. PC3, with PC1 explaining 38.8% of the total variance, while PC2 and PC3 explain 23.1 and 14.2%, respectively. Indeed, the three principal components together account for 76.1% of the total variability in the dataset. According to the plot (Figure 4), a clear separation of the samples can be observed according to the type of fruit and pulp or seed. PC1 represents a strong contrast between sugar and protein and mineral variables. For instance, it is positively correlated with total sugars (0.970), glucose (0.968), fructose (0.965), sucrose (0.930), C_ab_* (0.961) and moisture (0.919) (Table 7), indicating that this component captures these characteristics of the fruit pulps. In contrast, PC1 is negatively correlated with protein (−0.772), fat (−0.685), calcium (0.721), and ash content (−0.605) (Table 7), which are correlated with the fruit seeds. PC2 seems to be associated with organic acids and colour, showing high positive correlations with tartaric acid (0.858) (Table 7). On the other hand, PC2 is negatively correlated with succinic acid (−0.569), citric acid (−0.542), malic acid (−0.437) and formic acid (−0.443) as well as L (-0.790), hue angle (−0.920) and WI (−0.833). Regarding PC3, which explains less than 14.5% of the total variance, it is positively correlated with lactic acid (0.809), acetic acid (0.792) and citric acid (0.459) as well as colour parameters such as L (0.367) and WI (0.489), and has negative correlations with a lot of variables.

For PCA analysis, the carbohydrates, SDF, IDF, TDF and energy were not considered because these data were obtained only from pulps.

The plots (Figure 4) and the loadings (Table 7) reveal that the main sources of variability in the dataset are primarily related to the sugar content and moisture (PC1), followed by differences in tartaric acid and colour parameters (PC2), which demonstrates the diversity among the analysed samples (seed and pulp from three tropical fruits). As such, the PCA provides insight into the compositional dimensions that can distinguish the pulps and seeds of African fruits (velvet tamarind, African locust bean and baobab) and a framework for understanding the nutritional composition of these tropical food sources.

## 3. Discussion

The nutritional assessment of both pulps and seeds of *Dialium guineense* (velvet tamarind), *Parkia biglobosa* (African locust bean), and *Adansonia digitata* (baobab) revealed pronounced compositional differences, agreeing with previous reports on African fruits [8,13,19]. Regarding the colour of the fruits of these three African plant species (Figure 2 and Table 1), the data obtained are consistent with a previous study in which African locust bean pulp was described as bright yellow [20], while for baobab, Abdulwaliyu et al. [21] indicated that the pulp is covered with a skin bearing greyish-yellow hairs. Regarding *Dialium guineense* fruit pulp, it is referred to as red [22]. Moreover, the ΔE (Table 2) between the most contrasting samples (such as African locust bean pulp and velvet tamarind pulp, with a ΔE of 38) far exceeds the perception threshold of ΔE > 3.7 [23], confirming a clear visual differentiation (also verified by Figure 2). Overall, the data obtained (Table 1 and Table 2) shows the difference between each fruit and each part, i.e., pulp and seed.

Moisture content (Table 3) was assessed both for pulps and seeds of the three African fruits, with pulp exhibiting a significantly higher content (9.6 to 13%) compared to seeds (around 7%), reflecting their natural differences. The highest value for moisture was obtained for African locust bean fruit pulp, being higher than that reported by Gernmah et al. [20] (8.41%) and by Touré et al. [24] (9.78 to 12.31%), while the values observed for baobab (9.6%) are consistent with Angolan baobab pulp (12 to 13.8% reported by Monteiro et al. [13]), and Abiodun et al. [25] found 10.5% moisture content in a black velvet tamarind, being similar to the data (12%) presented in Table 3.

Moreover, regarding the ash, protein and fat contents of African locust bean pulp, our data (Table 3) presented lower values of ash and protein compared to Gernmah et al. [20] (ash 4.18% and protein 6.56%) but higher fat content (>1.80% obtained by Gernmah et al.) [20]. Considering baobab pulp, lower ash (2.1%) and protein contents (1.54%) were obtained (Table 3) compared to Monteiro et al. [13], with about 5%, and between 2.10% and 2.42%, respectively for ash and protein contents, while the same author states that baobab is a fruit characterized by a low protein and fat content; however, our data showed higher values of fat for baobab pulp (0.85%) compared to the other fruits. Velvet tamarind pulp also showed an ash content lower (2.04%) than the study carried out in black velvet tamarind pulp by Abiodun et al. [25] (12.6%). Additionally, regarding the protein and fat of velvet tamarind for both pulp and seeds, Arogba et al. [5] revealed lower values (61.3 g·kg^−1^ of protein in pulp, 148 g·kg^−1^ of protein in seeds, 70 g·kg^−1^ of fat in pulp and 60 g·kg^−1^ of fat in seeds). As such, this type of variation among parameters can be due to the sample’s origin and regions of production (i.e., soil type, edaphoclimatic conditions, fertilization). Despite the variations among pulps, protein and fat contents were significantly higher in the fruit’s seeds than in the pulp, which is not in accordance with Arogba et al. ‘s [5] data for lipid content regarding the pulp and seed of velvet tamarind.

The total dietary fibre analysis (Figure 3) revealed a significantly higher value for baobab pulp, being very similar to the data obtained by Monteiro et al. [13] for baobab pulp, it being essential to consider that our quantification used the same method as used in Monteiro et al. [13]. Despite the other two pulps showing lower values of TDF, they still delivered >30 g per 100 g of sample, exceeding the 25 g·day^−1^ of adequate intake for adults according to WHO [26].

The organic acid profile of both pulps and seeds of the three tropical fruits (Table 4) confirms the specific acid profile of these African fruits and with a generally lower acid load in seeds relative to their respective pulp. In this framework, velvet tamarind pulp had a high tartaric acid content (3.43%), being identified as the main contributor to its characteristic sweet-sour taste [25], which can also explain the intense reddish-brown colour (Figure 2 and Table 1) because tartaric acid forms coloured complexes with phenolics [27]. African locust bean pulp stands out with a higher succinic acid content (3.94%), followed by citric acid (2.8%) and formic acid (0.33%) (Table 4); in fact, both citric acid and formic acid showed higher values compared to the mean concentration reported by Touré et al. [24] for *Parkia biglobosa* pulp (1.1% for citric acid and 0.03% for formic acid) and by Gouveia-Nhanca et al. [28] for citric acid (0.13%). Baobab pulp displayed the highest citric acid (Table 4) concentration (3.24%), probably due to the fact that its characteristic taste is due to the presence of several organic acids [29], such as citric, tartaric, malic and succinic acid [30], which explain the values obtained in Table 4.

Regarding the sucrose, glucose, fructose and total sugar content of both pulps and seeds of the three African fruits analysed [18], it was possible that the highest concentration of sugar is present in pulp, and sucrose is the predominant carbohydrate, followed by glucose and fructose, showing a tendency of sucrose > glucose > fructose. The total sugar content of velvet tamarind pulp is 12%, which is higher than the value obtained by Abiodun et al. [25] for black velvet tamarind (41.89 g per 100 g). Moreover, according to the same study, velvet tamarind is valuable for its mineral and sugar contents, and it can be used as a sweetener in food products. Also, the sucrose, glucose, fructose pattern obtained follows a ratio of 5:4:3. African locust bean pulp, with almost 39% of total sugars, is by far the sweetest fruit analysed, with sucrose the major carbohydrate, being also reported by Gernmah et al. [20] as a fruit with a carbohydrate content of 67.30%. In fact, *Parkia biglobosa* fruit pulp is considered an energy-dense (48.4% carbohydrates and 305 Kcal per 100 g of sample—Table 5) sweetener very suitable for beverage formulations according to [31].

Considering the elemental composition of the three tropical fruits, overall, seeds have higher contents of Ca, Fe, P and S, while pulp is a better source of K (Table 6). For instance, baobab pulp showed similar values of Ca (3793 mg·kg^−1^) and lower values of K (37,528 mg·kg^−1^), P (10,805 mg·kg^−1^) and S (1960 mg·kg^−1^) considering the data reported by Monteiro et al. [13] from baobab samples from central inland in Namibe. Moreover, in Osman’s [32] study, Ca and Cu showed a higher content in seeds over fruit pulp and a higher K content in fruit pulp (as verified by our data, Table 6), and the Fe content was higher in pulp than in seed, but not according to our data. According to Asoiro et al. [7], velvet tamarind fruit pulp showed values of 0.5 g·kg^−1^ of P, 0.5 g·kg^−1^ of Ca and 0.1 g·kg^−1^ of Fe, being different from our data (Table 6), where Ca content > P content > Fe content. Regarding the seeds of African locust bean, a study carried out by Aborisade et al. [33] with African locust bean fruits from Nigeria showed values of Ca, K, P, S, Fe and Cu of 4446.2 mg·kg^−1^, 13,777.7 mg·kg^−1^, 3205.7 mg·kg^−1^, 15,361.7 mg·kg^−1^, 1266.8 mg·kg^−1^ and 3.7 mg·kg^−1^, respectively, showing a tendency of K > S > Ca > P > Fe > Cu, being different from the one observed in this study (Table 6): K > Ca > P > S > Fe > Cu, indicating variability that can be due to the cultivation practices and even the fruit’s origins.

The chemical composition of African locust bean fruit indicates that it is a good source of macro- and micro-nutrients. The bright yellow colour and high sugar content imparts sensory appeal to the pulp [20].

No toxic levels were quantified for the elements analysed (Hg < 6 mg·kg^−1^; Pb < 5 mg·kg^−1^; As < 3 mg·kg^−1^; Ni < 25 mg·kg^−1^ and Cd < 20 mg·kg^−1^), and considering amygdalin, a toxic compound present mostly in fruit seeds that can be metabolised to cyanide (a highly toxic compound) in the human body, was not detected in the fruit seeds. The method limit of detection for amygdalin was 6.34 mg per 100 g of sample, as such the seeds of the three African fruits had less than 6.34 mg amygdalin per 100 g of sample. The lethal amygdalin dosage is 0.5 mg·kg^−1^ of the person’s weight [34] and the ingestion of 500 mg amygdalin corresponds to 30 mg cyanide [35].

The PCA confirms that sugars and even moisture define pulps, whereas proteins, lipids and minerals define seeds, with each fruit species being distinct, and these plots of compositional dimensions provide a rapid visual tool to distinguish and valorise the pulp and seed of velvet tamarind, African locust bean and baobab (Figure 4).

## 4. Materials and Methods

### 4.1. Experimental Design

The fruits were hand harvested by the supplier after they had ripened in trees of *Dialium guineense* Willd, *Parkia biglobosa* Jacq. and *Andansonia digitata* L. and were purchased (800 g–1000 g of fruits) in a Guinea-Bissau local market, placed in hermetic recipients and transported by plane (at room temperature in the hermetic recipients sealed to prevent gas exchange) to INIAV, Oeiras, Portugal. The seeds were manually separated from pulp in the laboratory and allowed to dry (MMM Venticell, München, Germany) at 60 °C until a constant weight. Seed and pulp samples were then ground to a powder in a laboratory mill (IKA, A10, Staufen, Germany) and sieved to particles less than 500 µm.

A physicochemical characterization of the fruit pulps and seeds was performed for colour, moisture, ash, protein, fat, total dietary fibre, free sugar and organic acid profiles, and elemental analysis. Total carbohydrate and energy contents were calculated only for pulps. All determinations were conducted in triplicate unless indicated otherwise.

All reactants and solvents were purchased from Merck (Darmstadt, Germany) or Panreac (Barcelona, Spain) and were of analytical or chromatographic grade. Standards were from Sigma (Burlington, MA, USA). The solutions were prepared with ultrapure water (model Milli-Q 7000, Millipore SAS, Molsheim, France). The mobile phases were vacuum filtered (pump GAST, model DOAP104-BN, Benton Harbor, MI, USA) with a 0.45 μm nylon membrane followed by ultrasonic degassing (Bransonic, Branson 5200, Branson, MO, USA) for at least 25 min before use.

### 4.2. Physicochemical Characterisation

#### 4.2.1. Colourimetric Properties

Colour determination was achieved with a colourimeter (Minolta CR-300, Osaka, Japan) calibrated with a white tile standard (L* = 97.10; a* = 0.08; b* = 1.80) to measure the CIE L*, a*, b* parameters. The measuring was performed by inserting the probe into the sample powder and the measurement was performed 5 times per sample.

Chroma index (C*_ab_), whiteness index (WI), hue angle, and total colour difference (ΔE) were calculated using the following equations:

C*ab = (a*^2^ + b*^2^)^1/2^ (1)

WI = 100 − [(100 − L*)^2^ + a*^2^ + b*^2^] ^1/2^ (2)

Hue angle = arctan (b*/a*)(3)

ΔE_1/2_ = [(L*_1_ − L*_2_) ^2^ + (a*_1_ − a*_2_)^2^ + [(b*_1_ − b*_2_)^2^] ^1/2^ (4)

where ΔE_1/2_ represents the colour difference between sample 1 and sample 2 [36].

#### 4.2.2. Macro Characterization

The moisture determination was performed in percentage by a gravimetric method, consisting of drying 1.0 g of the sample at 103 °C in an oven (Memmert, U50, Schwabach, Germany) until a constant weight as described by Monteiro et al. [13].

The ash content in percentage was determined using a gravimetric method after incineration of 1.0 g of the sample at 550 °C for six hours in a muffle furnace (Thermolyne Muffle Furnace, model 48000, Dubuque, IA, USA) as described by Pereira et al. [37].

The total nitrogen content was determined in percentage by weighing 0.5 g of the sample and using the Kjeldahl method with hot digestion (digestor Foss, Tecator 2020 Digestor, Hillerød, Denmark) and automatic distillation (Foss, 2300 Kjeltec analyser unit, Hillerød, Denmark). The crude protein content was calculated in percentage by multiplying the determined nitrogen content by the 6.25 conversion factor, as in Nicolai et al. [38].

The fat content of fruit pulps and seeds was determined by weighing 5.0 g of dry sample into a Soxhlet extraction cellulose cartridge. The cartridge was then placed in a Soxhlet extractor with a distillation flask containing 200 mL ether petroleum and was extracted continuously for six hours. The solvent was eliminated by evaporation at 30 °C in an oven (WRC Binder, Tuttlingen, Germany), and the fat content was quantified by weighing as described by Nicolai et al. [38].

The soluble and insoluble dietary fibre were determined by an enzymatic gravimetric procedure using 1 g of the fruit pulp sample (previously dried) according to the Megazyme fibre kit procedure (Megazyme kit K-TDFR, José Manuel Gomes dos Santos, Lisbon, Portugal) and as described by Nicolai et al. [38]. The fruit pulp total dietary fibre was determined as the sum of the soluble and the insoluble dietary fibres.

The free sugar profile was determined in the fruit pulps and seeds as previously described by Lageiro et al. [18].

The organic acid profile was determined in fruit pulps and seeds (previously dried) by HPLC-PDA (photodiode array detector). The organic acid extraction was performed by homogenisation of 1.0 g of the sample in phosphate buffer (0.05 M, pH 2.8) in a polytron (Ika, Ultra-Turrax T25, Staufen, Germany) as described by Pereira et al. [39].

The HPLC-PDA (Waters Alliance 2695 with PDA 996, Waters, Milford, MA, USA) separation used a Rezex™ ROA column (Phenomenex, Torrance, CA, USA) as described by Cartas et al. [40], kept at 25 °C, and used a 0.01 M sulfuric acid mobile phase for 30 min at a flow rate of 0.5 mL.min^−1^ in isocratic mode, and 10 μL injection volumes. The organic acid identification was conducted at 210 nm by comparison of the organic acid standards’ retention time and UV spectra. The quantification of organic acids was performed by peak area comparison to a calibration mixture of organic acid standards comprising acetic, citric, formic, lactic, malic, oxalic, succinic and tartaric acids in the range of 20 to 2000 μg/mL.

The total carbohydrate content was calculated in percentage only in fruit pulp via Equation (5) as described by Monteiro et al. [13], considering a dry weight basis.

Carbohydrate (%) = 100 − [moisture (%) + ash (%) + protein (%) + fat (%) + fibre (%)](5)

The energy was calculated in kcal per 100 g (dry weight basis), only in fruit pulp via Equation (6) as described by Panda et al. [41].

Energy = 4 × Protein + 4 × Carbohydrate + 9 × Fat + 2 × Fibre + 3 × Total organic acid(6)

where the energy is in Kcal per 100 g of dry weight sample and the other determinations are in g per 100 g of sample.

#### 4.2.3. Toxic Amygdaline Content

The toxicological content of amygdalin was determined by HPLC-PDA in dry fruit seeds and the extraction of the amygdaline from the samples was performed by ethanol hot extraction at 78.5 °C under reflux as described by Bolarinwa et al. [42,43], with a sample mass of 2.0 g and 10 mL extraction volume.

The HPLC determination of amygdalin was performed with Waters equipment (Milford, MA, USA). It used a Waters Spherisorb ODS2 separation column, maintained at 40 °C, a PDA detector (Waters Alliance 2695, 2996 PDA, Milford, MA, USA), a mobile phase of 25% ethanol and 75% water, at a flow rate 1 mL/min through the reverse phase C18 column. Samples were kept at 5 °C until injection and the injection volume was 20 µL. Identification was made by retention time and spectra comparisons between the sample and standard chromatograms with integration at 200 nm. Quantification was performed by linear regression between the peak area and standard concentration with the equation: peak area = 9991.6 × amygdalin (mg L^−1^) + 41,331.7 and R^2^ = 0.9998, in a range from 1 to 200 mg amygdalin mL^−1^. The method limit of detection was 6.34 mg amygdalin per 100 g sample and the limit of quantification was 19.22 mg amygdalin per 100 g of sample.

#### 4.2.4. Elemental Analysis

The samples were manually peeled and pulps and seeds were separated and dried at 60 °C until a constant weight. After drying, the samples were ground into a fine powder using an agate mortar and the contents of Ca, K, P, S, Fe and Cu were quantified using an X-ray analyser (Thermo Scientific™, Niton™ XL3t 950 He GOLDD+, Waltham, MA, USA) as described by Fernandes et al. [44].

### 4.3. Statistical Analysis and Principal Component Analysis

Statistical analysis was performed using R software version 4.4.2. (GNU General Public License, Boston, MA, USA). One-way analysis of variance (ANOVA) was applied and when significant differences were detected (*p* < 0.05, i.e., with 95% confidence level), Scheffé’s post hoc test was performed for all the parameters analysed to identify statistically distinct groups among the different samples. Additionally, a principal component analysis (PCA) was performed, being generally applied as the first tool to analyse multivariate data, to explore relationships among samples based on their compositional profiles, being very efficient in revealing the main contrasting regions in the plot. As such, the data from the three main principal components were plotted, considering all the different parameters analysed. The loadings were analysed, providing interpretation by showing positive and negative values to correlate or not with the variables.

## 5. Conclusions

The fruits from *Dialium guineense* Willd, *Parkia biglobosa* Jacq. and *Andansonia digitata* L. plants showed different characteristics, namely regarding their contents of sugars, protein, fat, fibre and organic acid profile, offering natural sweetening (African locust bean), a low-calorie option (baobab) or high protein content (velvet tamarind). Seeds were found to be good protein, lipid, Ca, P, Fe, and S sources, which can lead to a development of flours with functional characteristics. Moreover, the pulps of the three fruits showed high contents of K, being a good source for humans. Both pulp and seeds were confirmed nutritional safe, without heavy metals and non-detectable amygdalin. Moreover, this study provides information for health professionals like nutritionists and food policymakers in Africa to encourage the population to consume these tropical fruits and promote the conservation of the *Dialium guineense* Willd, *Parkia biglobosa* Jacq. and *Andansonia digitata* L. species. Finally, the findings of this study are intended to fill a literature gap regarding the lack of information about fruits and seeds of Guinea-Bissau origin, the nutritional compositions of the fruits of the velvet tamarind, African locust bean and baobab.

## Figures and Tables

**Table 1:** Colourimetric parameters (L*, a*, b*, whiteness index (WI), C_ab_* (chroma/saturation) and hue angle) of fruit pulps and seeds of *Dialium guineense* Willd (velvet tamarind), *Parkia biglobosa* Jacq. (African locust bean) and *Andansonia digitata* L. (baobab). The data are presented as the means ± SDs (*n* = 5). ANOVA, *p* < 0.05, was performed for each parameter. Different letters express significant differences between each type of fruit (a, b), with a for the highest values.

Type	Fruit	L*	a*	b*	WI	C_ab_*	Hue Angle
Pulp	Velvet tamarind	48.81 ± 0.48 ^cd^	18.6 ± 0.39 ^a^	22.97 ± 0.17 ^b^	40.89 ± 0.52 ^c^	29.56 ± 0.27 ^b^	51.02 ± 0.64 ^f^
	African locust bean	79.87 ± 0.11 ^a^	3.08 ± 0.03 ^d^	38.4 ± 0.1 ^a^	56.54 ± 0.06 ^ab^	38.52 ± 0.1 ^a^	85.41 ± 0.05 ^a^
	Baobab	73.09 ± 0.08 ^ab^	7.82 ± 0.03 ^b^	21.14 ± 0.03 ^c^	64.9 ± 0.07 ^a^	22.54 ± 0.03 ^c^	69.69 ± 0.07 ^d^
Seed	Velvet tamarind	60.35 ± 6.81 ^bc^	5.09 ± 0.13 ^c^	15.51 ± 0.08 ^e^	56.88 ± 6.42 ^ab^	16.32 ± 0.11 ^e^	71.85 ± 0.34 ^c^
	African locust bean	72.81 ± 0.18 ^ab^	2.94 ± 0.09 ^d^	19.71 ± 0.13 ^d^	66.29 ± 0.08 ^a^	19.93 ± 0.12 ^d^	81.51 ± 0.27 ^b^
	Baobab	45.2 ± 0.3 ^d^	6.94 ± 0.09 ^b^	11.88 ± 0.21 ^f^	43.5 ± 0.25 ^bc^	13.76 ± 0.19 ^f^	59.67 ± 0.53 ^e^

**Table 2:** Colour difference (ΔE) between fruits (pulps and seeds) of *Dialium guineense* Willd (velvet tamarind), *Parkia biglobosa* Jacq. (African locust bean) and *Andansonia digitata* L. (baobab). Each value represents the total colour difference between fruits and parts (pulp and seed), carried out based on the average of the colourimetric parameters.

ΔE	ΔE Pulp	ΔE Seed	ΔE Pulp/Seed
Velvet tamarind/African locust bean	37.99	13.33	28.84
Velvet tamarind/Baobab	26.63	15.69	16.49
African locust bean/Baobab	19.13	28.98	5.10

**Table 3:** Evaluation of fruit pulps and seeds of *Dialium guineense* Willd (velvet tamarind), *Parkia biglobosa* Jacq. (African locust bean) and *Andansonia digitata* L. (baobab), regarding moisture (%), ash (%), protein (%, dry weight) and fat (%, dry weight) content. The data are presented as the means ± SDs (*n* = 3), ANOVA *p* < 0.05. Different letters express significant differences between each type of fruit for each parameter (a, b), with a for the highest values.

Type	Fruit	Moisture (%)	Ash (%)	Protein (%)	Fat (%)
Pulp	Velvet tamarind	12.14 ± 0.32 ^b^	2.04 ± 0.07 ^c^	2.62 ± 0.11 ^d^	0.27 ± 0.00 ^d^
	African locust bean	13.04 ± 0.15 ^a^	2.21 ± 0.01 ^b^	2.29 ± 0.03 ^d^	0.80 ± 0.00 ^d^
	Baobab	9.60 ± 0.24 ^c^	2.24 ± 0.12 ^b^	1.54 ± 0.07 ^d^	0.85 ± 0.00 ^d^
Seed	Velvet tamarind	7.52 ± 0.30 ^d^	2.38 ± 0.10 ^b^	19.89 ± 1.00 ^c^	4.53 ± 0.00 ^c^
	African locust bean	7.09 ± 0.22 ^d^	2.41 ± 0.10 ^b^	28.41 ± 0.07 ^a^	18.85 ± 0.00 ^b^
	Baobab	7.54 ± 0.04 ^d^	2.52 ± 0.07 ^a^	23.36 ± 0.35 ^b^	21.49 ± 0.00 ^a^

**Table 4:** Acetic, citric, formic, lactic, malic, oxalic, succinic and tartaric acid contents (%, dry weight) of fruit pulps and seeds of *Dialium guineense* Willd (velvet tamarind), *Parkia biglobosa* Jacq. (African locust bean) and *Andansonia digitata* L. (baobab). The data are presented as the means ± SDs (*n* = 3), ANOVA *p* < 0.05. Different letters express significant differences between each type of fruit for each parameter (a, b), with a for the highest values.

Organic Acid (%)	Velvet Tamarind		African Locust Bean		Baobab	
	Pulp	Seed	Pulp	Seed	Pulp	Seed
Acetic	0.038 ± 0.01 ^b^	<0.016	<0.016	<0.016	2.501 ± 0.00 ^a^	<0.016
Citric	<0.007	0.939 ± 0.12 ^b^	2.826 ± 0.88 ^a^	1.000 ± 0.12 ^b^	3.243 ± 0.41 ^a^	0.776 ± 0.01 ^b^
Formic	<0.076	<0.076	0.326 ± 0.33 ^a^	1.038 ± 0.11 ^a^	0.995 ± 0.00 ^a^	0.230 ± 0.09 ^a^
Lactic	0.113 ± 0.02 ^a^	<0.014	<0.014	<0.014	0.351 ± 0.00 ^a^	<0.014
Malic	0.579 ± 0.37 ^c^	4.321 ± 0.18 ^a^	1.961 ± 0.59 ^b^	0.954 ± 0.08 ^c^	0.970 ± 0.23 ^bc^	0.349 ± 0.01 ^c^
Oxalic	0.233 ± 0.11 ^c^	0.203 ± 0.01 ^c^	0.477 ± 0.08 ^b^	0.078 ± 0.01 ^c^	<0.004	0.696 ± 0.01 ^a^
Succinic	<0.050	1.520 ± 0.02 ^b^	3.937 ± 0.52 ^a^	0.866 ± 0.09 ^bc^	0.487 ± 0.00 ^c^	0.754 ± 0.02 ^bc^
Tartaric	3.43 ± 1.42 ^a^	<0.005	<0.005	<0.005	<0.005	<0.005

**Table 5:** Carbohydrates (%, dry weight basis) and energy of fruit pulps were calculated by Kcal per 100 g of dry weight for *Dialium guineense* Willd (velvet tamarind), *Parkia biglobosa* Jacq. (African locust bean) and *Andansonia digitata* L. (baobab). Data are presented as the means ± SDs (*n* = 3). ANOVA, *p* < 0.05, performed for carbohydrate contents. Different letters express significant differences between each type of fruit (a, b), with a for the highest values.

Type	Fruit	Carbohydrates (%)	Energy (Kcal per 100 g of Dry Weight)
Pulp	Velvet tamarind	45.0 ± 0.48 ^b^	282.0 ± 7.3 ^b^
	African locust bean	48.4 ± 0.14 ^a^	305.0 ± 6.6 ^a^
	Baobab	34.2 ± 0.31 ^c^	279.3 ± 3.2 ^c^

**Table 6:** Calcium, potassium, phosphorus, sulfur, iron and copper content (mg of mineral element per 100 g of dry weight basis) of fruit pulps and seeds of *Dialium guineense* Willd (velvet tamarind), *Parkia biglobosa* Jacq. (African locust bean) and *Andansonia digitata* L. (baobab). Data are presented as the means ± SDs (*n* = 3), ANOVA, *p* < 0.05. Different letters express significant differences between each type of fruit for each parameter (a, b), with a for the highest values.

Type	Fruit	Ca	K	P	S	Fe	Cu
Pulp	Velvet tamarind	66.6 ± 4.8 ^e^	857.9 ± 13.0 ^f^	32.2 ± 4 ^d^	15.7 ± 4.6 ^e^	3.1 ± 0.3 ^d^	1.8 ± 0.3 ^b^
	African locust bean	107.2 ± 5.6 ^d^	3263 ± 9.3 ^a^	79.7 ± 2 ^b^	26.6 ± 0.7 ^de^	5.5 ± 0.4 ^bc^	3.7 ± 0.8 ^a^
	Baobab	310.4 ± 6.6 ^c^	2777 ± 14.2 ^b^	25.5 ± 5.5 ^d^	30 ± 1.1 ^d^	4.4 ± 1.0 ^cd^	1.8 ± 0.1 ^b^
Seed	Velvet tamarind	406.4 ± 3.7 ^a^	1305 ± 6.6 ^e^	171.9 ± 8.5 ^c^	280.9 ± 2.9 ^c^	6.3 ± 0.7 ^b^	1.6 ± 0.4 ^b^
	African locust bean	257.2 ± 3.1 ^b^	1737 ± 16.4 ^d^	183.5 ± 5.6 ^c^	2138 ± 6.6 ^a^	<3.5	1.5 ± 0.2 ^b^
	Baobab	327.5 ± 8.3 ^c^	2265 ± 11.3 ^c^	768.7 ± 7 ^a^	314.7 ± 5.3 ^b^	10.9 ± 0.2 ^a^	2.7 ± 0.3 ^ab^

**Table 7:** Correlations between variables with the principal components (PC1, PC2, PC3 and PC4).

Variable	PC1	PC2	PC3	PC4
Fat	−0.685	−0.222	−0.516	0.399
Protein	−0.772	−0.370	−0.443	−0.042
Total sugar	0.970	−0.121	−0.166	0.018
Sucrose	0.930	−0.165	−0.209	−0.009
Glucose	0.968	−0.105	−0.141	0.060
Fructose	0.965	−0.105	−0.156	−0.001
Oxalic	0.142	0.143	−0.820	0.313
Citric	0.498	−0.542	0.459	0.365
Tartaric	0.162	0.858	0.051	−0.245
Lactic	0.046	0.189	0.809	0.352
Succinic	0.705	−0.569	−0.378	−0.100
Formic	−0.111	−0.443	0.236	0.239
Acetic	0.007	−0.020	0.792	0.408
Malic	0.049	−0.437	0.000	−0.775
Moisture	0.919	0.373	0.058	0.023
Ash	−0.605	−0.495	−0.308	0.295
Ca	−0.721	−0.461	0.111	−0.079
K	−0.474	−0.446	−0.100	0.034
Fe	−0.065	−0.093	−0.018	−0.920
Cu	0.648	−0.114	−0.463	0.341
L*	0.438	−0.790	0.367	−0.123
a*	0.031	0.965	0.184	−0.113
b*	0.959	−0.169	0.016	0.008
WI	−0.092	−0.833	0.489	−0.193
C_ab_*	0.961	0.095	0.036	−0.024
Hue angle	0.321	−0.920	−0.007	−0.020

## Data Availability

The original contributions presented in this study are included in the article. Further inquiries can be directed to the corresponding authors.
